# First-Pass Meconium Samples from Healthy Term Vaginally-Delivered Neonates: An Analysis of the Microbiota

**DOI:** 10.1371/journal.pone.0133320

**Published:** 2015-07-28

**Authors:** Richard Hansen, Karen P. Scott, Shoaib Khan, Jenny C. Martin, Susan H. Berry, Matthew Stevenson, Augusta Okpapi, Michael J. Munro, Georgina L. Hold

**Affiliations:** 1 Department of Neonatology, Aberdeen Maternity Hospital, Aberdeen, United Kingdom; 2 Gastrointestinal Research Group, University of Aberdeen, Aberdeen, United Kingdom; 3 Rowett Institute of Nutrition and Health, University of Aberdeen, Aberdeen, United Kingdom; University British Columbia, CANADA

## Abstract

**Background:**

Considerable effort has been made to categorise the bacterial composition of the human gut and correlate findings with gastrointestinal disease. The infant gut has long been considered sterile at birth followed by rapid colonisation; however, this view has recently been challenged. We examined first-pass meconium from healthy term infants to confirm or refute sterility.

**Methods:**

Healthy mothers were approached following vaginal delivery. First-pass meconium stools within 24 hours of delivery were obtained from healthy, breastfed infants with tight inclusion/exclusion criteria including rejecting any known antibiotic exposure - mother within 7 days preceding delivery or infant after birth. Stools were processed in triplicate for fluorescent in-situ hybridisation (FISH) with 16S rRNA-targeted probes including *Bifidobacterium*; *Bacteroides*-*Prevotella*; *Lactobacillaceae*/*Enterococcaceae*; *Enterobacteriaceae*; *Streptococcaceae*; *Staphylococcaceae* and *Enterococcaceae*. Absolute counts of all bacteria and proportional identification of each bacterial group were calculated. Confirmation of bacterial presence by PCR was undertaken on FISH-positive samples.

**Results:**

The mothers of 31 newborn infants were recruited, 15 met inclusion/exclusion criteria and provided a sample within 24 hours of birth, processed in the lab within 4 hours. All babies were 37–40 weeks gestation. 8/15 were male, mean birth weight was 3.4kg and mean maternal age was 32 years. Meconium samples from 10/15 (66%) infants had evidence of bacteria based on FISH analysis. Of these, PCR was positive in only 1. Positive FISH counts ranged from 2.2 - 41.8 x 10^4^ cells/g with a mean of 15.4 x 10^4^ cells/g. (The limit of detection for automated counting is 10^6^ cells/g). Cell counts were too low to allow formal diversity analysis. Amplification by PCR was not possible despite positive spiked samples demonstrating the feasibility of reaction. One baby was dominated by *Enterobacteriaceae*. The others contained 2-5 genera, with *Bifidobacterium*, *Enterobacteriaceae*, *Enterococcaceae* and *Bacteroides*-*Prevotella* the most prevalent. There was no association between bacterial counts and rupture of membrane duration, time to passage of meconium or time to lab.

**Conclusion:**

This study provides evidence that low numbers of bacteria are present in first-pass meconium samples from healthy, vaginally-delivered, breastfed term infants. Only two-thirds of meconium samples had detectable bacteria, though at levels too low for automated counting or for reliable confirmation by PCR. This study suggests that gut bacterial colonisation is extremely limited at birth and occurs rapidly thereafter.

## Introduction

There has been a recent explosion of interest in the potential role of resident gut bacteria (the gut microbiota) in the causation or alleviation of human disease, however comparatively little attention has been paid to the process of early life colonisation. The long-standing dogma of gut sterility at birth has recently been challenged by suggestions that early meconium samples [1], the placenta [2] and even umbilical cord blood [3] harbour evidence of bacteria.

The simple question of whether or not the gut is sterile at birth is a fundamental one in understanding human microbial colonisation, particularly as the gut harbours the most diverse bacterial community of the human body [4]. Data gathered from across different species have repeatedly demonstrated microbial transfer from mother to infant, supporting the critical nature of this physiological process [5]. Extending positive microbial findings however from the placenta [2], amniotic sac [6], umbilical blood [3] or breastmilk [7] to the gut of the fetus requires targeted study to address the paradigm of gut sterility at birth. The first question that needs to be robustly answered in the ongoing study of bacterial colonisation therefore is the sterility, or otherwise, of the gut at birth. With this in mind, a short, targeted study in healthy, term, vaginally-delivered, breastfed infants was undertaken to look for evidence of bacteria in first-pass meconium samples using two distinct molecular techniques. We elected to focus on fluorescent in-situ hybridisation (FISH) rather than the more prevalent amplicon sequencing methodology for two main reasons: firstly, amplicon sequencing is a qualitative and not quantitative method, so the absolute abundance of bacteria is not known; and secondly, in amplicon sequencing bacteria are identified based on DNA, not viable cells. Additionally, any DNA-based analysis of samples containing low levels of bacteria has to be carefully assessed for the possibility of contamination during processing confounding the results [8]. These are the reasons we specifically chose to utilise FISH for our work- it is quantitative and it allows visualisation of cells, inferring the presence of viable organisms in the sample studied.

## Methods

Healthy mothers following a normal vaginal delivery at term in Aberdeen Maternity Hospital were invited to participate in the study. Primigravida and parous mothers were considered equally. The first meconium nappy, passed within 24 hours of delivery, was collected and processed in the laboratory within 4 hours. Although the method of feeding was not thought to be of significance during this short period after birth, only infants exclusively breastfed up to the point of first meconium were included.

Tight inclusion and exclusion criteria were applied to approximate normal physiology as closely as possible within a hospital setting. Inclusion criteria: healthy mother; healthy pregnancy; term at delivery (37–42 weeks inclusive); appropriate weight for gestational age (between 10^th^- 90th centiles); vaginal delivery; healthy infant; breastfed only to point of sample collection. Exclusion criteria: significant background maternal health concerns; significant maternal health issues during pregnancy; perinatal antibiotic exposure (mother within 7 days preceding delivery or infant after birth); premature delivery (<37 weeks); post-term delivery (>42 weeks); small weight for gestational age (weight <10th centile); large weight for gestational age (weight >90th centile); Caesarean section delivery; instrumental delivery; prolonged rupture of membranes prior to delivery (>24 hours); meconium passed in liquor; maternal pyrexia>38°C during labour; neonatal health concerns sufficient to warrant admission to neonatal unit; formula milk fed at any point prior to sample. The period prior to delivery within which maternal antibiotic exposure was deemed an exclusion criterion (7 days) was decided on a pragmatic basis. Data on inclusion and exclusion criteria were collected for each recruit on a study proforma sheet and cross-checked during analysis.

Meconium samples were collected using routine “clean” technique within the hospital setting (researcher’s hands washed, non-sterile gloves applied, sterile spatula used to retrieve sample) into a sterile universal container, placed immediately into a fridge in the maternity unit then transferred to the laboratory and processed within 4 hours of collection. This same initial approach is used routinely in the collection of multiple clinical samples for hospital microbial analysis, is in keeping with standard practice. Samples were then received into Category 2 microbiology laboratories and processed.

Firstly, 0.5g samples were taken from the meconium specimens and placed into lysing matrix tubes from the FastDNA SPIN Kit for Soil (MP Biomedical, Ohio, USA) then stored at -80°C until DNA extraction was performed in batches using the same kit within 8 weeks of collection. Secondly, a 0.5g sample was fixed in a 1/40 solution for fluorescent in-situ hybridisation (FISH) following the stool processing protocol [9]. Briefly, 1–2ml PBS/30% glycerol and a few glass beads were added and the mix vortexed, until the meconium was suspended, before dilution in further PBS/30% glycerol to a final volume of 3.3ml. 1ml of supernatant was transferred into 3ml of 4% paraformaldehyde, mixed by hand and stored for FISH analysis.

16S rRNA-targeted fluorescent probes were used to detect the predominant groups of human faecal bacteria. Absolute counts of all bacteria and proportional identification for each bacterial group were calculated. The probes used were Eub338 (total bacterial count), Bif164 (*Bifidobacterium* genus), Bac303 (*Bacteroides*-*Prevotella* group), LAB158 (*Lactobacillaceae* and *Enterococcaceae*), EntD (*Enterobacteriaceae*), *Streptococcaceae* (Strep), *Staphylococcaceae* (Staphy) and specifically *Enterococcaceae* (ENC221). These probes have all been validated previously and hybridisation was carried out using standard methods [10–12]. Slight cross-hybridisation between ENC221 and *Streptococcus* species was observed. The low bacterial load in the samples meant that automated counting could not be done and each field of view had to be examined and counted manually.

Universal bacterial quantitative real-time PCR was performed on FISH positive samples as described previously [13]. Briefly, standard curves consisted of ten-fold dilution series of amplified bacterial 16S rRNA genes from reference strains. The abundance of 16S rRNA gene copies was determined from standard curves. Due to the relatively low abundance of bacterial DNA in meconium samples, 25ng of DNA was used per reaction.

Finally, we wanted to compare total bacterial counts against a clinical composite of the period during which colonisation may have commenced if the uterine environment is presumed sterile, hence we defined the “meconium colonisation interval” (MCI) as the total time from rupture of membrane to time of birth, plus time to passage of meconium, plus time to laboratory processing and have used this in analysis.

### Ethics Statement

Ethical approval was granted by North of Scotland Research Ethics Service (10/S0801/16) and written informed consent was obtained from the parents of all subjects.

## Results

The mothers of 31 infants were identified, approached and consented for the study immediately post-partum. Of these, 18 ultimately met the inclusion/exclusion criteria and 15 provided a meconium sample within 24 hours, which was subsequently collected and processed in the laboratory within 4 hours. All babies were 37–40 weeks gestation. 8/15 were male, mean birth weight was 3.4kg and mean maternal age was 32 years. The final cohort (n = 15) is described in Tables 1 and 2.

**Table 1:** Maternal details for final cohort.

Maternal Age	Maternal Health	Health In Pregnancy	Alcohol in Pregnancy	Smoking in Pregnancy	Illicit Drug Use in Pregnancy	Rupture Of Membranes	Meconium in Liquor	Method of Delivery	Pyrexia in Labour	Rupture of Membranes >24h	Antibiotics During Pregnancy	Antibiotics During Labour	Antibiotics Postnatal	Antenatal Steroids
32	Healthy	Healthy	No	No	No	Spontaneous	No	Standard vaginal vertex	No	No	No	No	No	No
31	Healthy	Healthy	No	No	No	Spontaneous	No	Standard vaginal vertex	No	No	No	No	No	No
27	Healthy	Healthy	No	No	No	Spontaneous	No	Standard vaginal vertex	No	No	No	No	No	No
32	Healthy	Healthy	No	Yes	No	Assisted	No	Standard vaginal vertex	No	No	No	No	No	No
26	Healthy	Healthy	No	No	No	Spontaneous	No	Standard vaginal vertex	No	No	No	No	No	No
27	Healthy	Healthy	No	No	No	Spontaneous	No	Standard vaginal vertex	No	No	No	No	No	No
32	Healthy	Healthy	No	No	No	Spontaneous	No	Standard vaginal vertex	No	No	No	No	No	No
29	Healthy	Healthy	No	No	No	Spontaneous	No	Standard vaginal vertex	No	No	No	No	No	No
37	Healthy	Healthy	No	No	No	Spontaneous	No	Standard vaginal vertex	No	No	No	No	No	No
44	Healthy	Healthy	No	No	No	Spontaneous	No	Standard vaginal vertex	No	No	No	No	No	No
29	Healthy	Healthy	No	No	No	Spontaneous	No	Standard vaginal vertex	No	No	No	No	No	No
39	Healthy	Healthy	No	No	No	Spontaneous	No	Standard vaginal vertex	No	No	No	No	No	No
27	Healthy	Healthy	No	No	No	Spontaneous	No	Standard vaginal vertex	No	No	No	No	No	No
31	Healthy	Healthy	No	No	No	Assisted	No	Standard vaginal vertex	No	No	No	No	No	No
35	Healthy	Healthy	No	No	No	Spontaneous	No	Standard vaginal vertex	No	No	No	No	No	No

**Table 2:** Neonatal details for final cohort.

Baby Sex	Gestation	Birth Weight	APGAR at 1 minute	APGAR at 5 minutes	Resuscitation	Admission to NNU	Postnatal Antibiotics	Feeding	ROM Time (minutes)	Birth To Meconium Time (minutes)	Meconium To Lab Time (minutes)
Female	38	2.9	9	9	None/Stimulation/Facial O2	No	No	Attempt at/successful breastfeeding	3	202	83
Female	40	3.3	9	9	None/Stimulation/Facial O2	No	No	Attempt at/successful breastfeeding	51	334	84
Male	37	2.9	9	9	None/Stimulation/Facial O2	No	No	Attempt at/successful breastfeeding	12	346	85
Male	37	2.8	9	9	None/Stimulation/Facial O2	No	No	Attempt at/successful breastfeeding	698	1352	50
Female	40	3.3	9	9	None/Stimulation/Facial O2	No	No	Attempt at/successful breastfeeding	33	277	36
Female	39	3.6	9	9	None/Stimulation/Facial O2	No	No	Attempt at/successful breastfeeding	73	377	30
Male	40	4.1	9	9	None/Stimulation/Facial O2	No	No	Attempt at/successful breastfeeding	17	238	40
Male	40	3.5	9	9	None/Stimulation/Facial O2	No	No	Attempt at/successful breastfeeding	49	185	15
Male	39	3.4	9	9	None/Stimulation/Facial O2	No	No	Attempt at/successful breastfeeding	9	483	180
Female	40	3.4	8	9	None/Stimulation/Facial O2	No	No	Attempt at/successful breastfeeding	6	442	120
Female	40	3.8	9	9	None/Stimulation/Facial O2	No	No	Attempt at/successful breastfeeding	224	256	30
Male	39	3.2	9	10	None/Stimulation/Facial O2	No	No	Attempt at/successful breastfeeding	225	495	30
Male	39	3.1	9	9	None/Stimulation/Facial O2	No	No	Attempt at/successful breastfeeding	333	527	120
Female	39	3.5	9	9	None/Stimulation/Facial O2	No	No	Attempt at/successful breastfeeding	390	495	180
Male	40	3.8	9	10	None/Stimulation/Facial O2	No	No	Attempt at/successful breastfeeding	18	442	150

**Fig 1:**
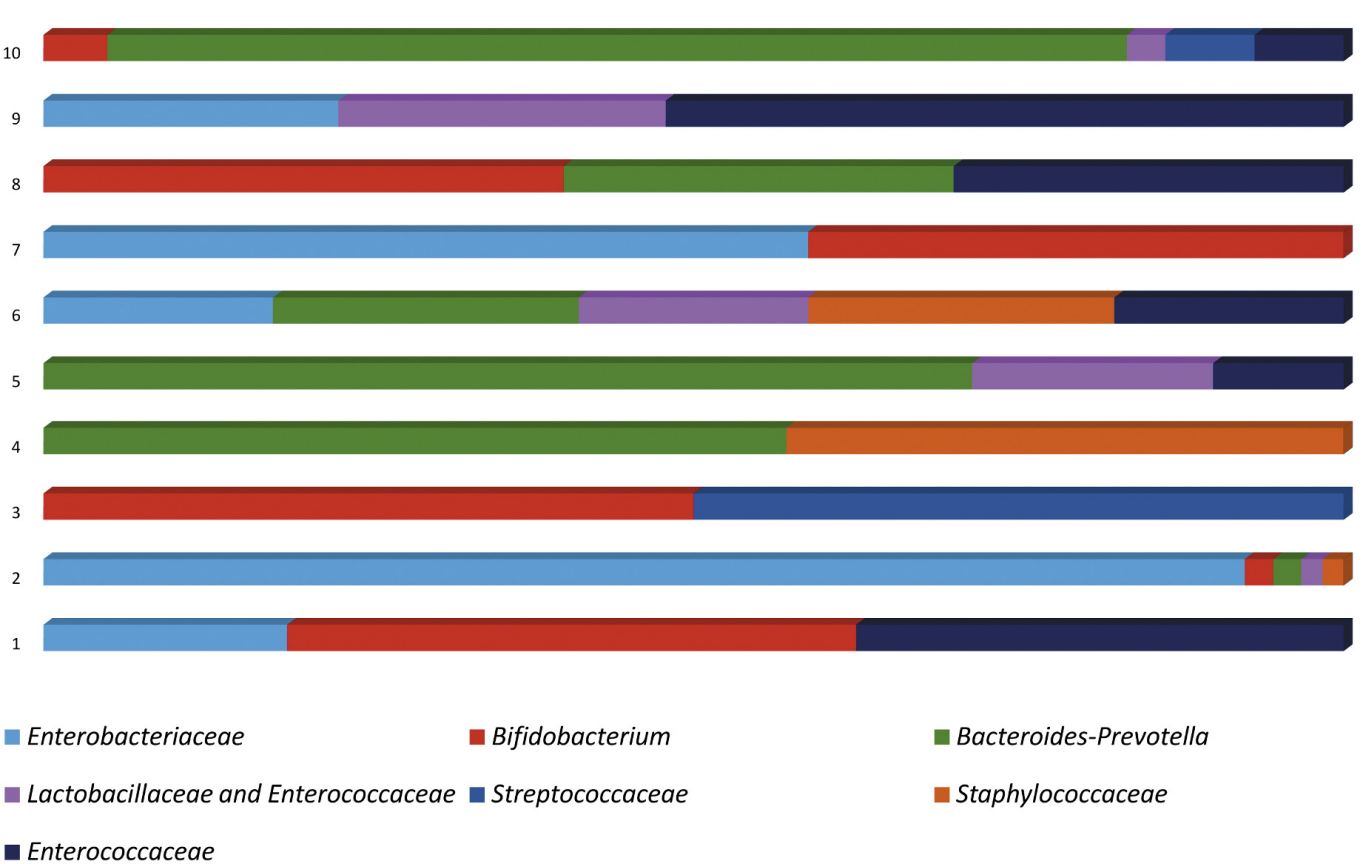
Diagrammatic indication of bacterial composition in FISH-positive meconium samples based on proportions of specific bacterial groups relative to the total bacterial count.

Following FISH analysis, 10/15 (66%) infants had evidence of bacteria present. Positive FISH counts ranged from 2.2 to 41.8 x 10^4^ cells/g, with the mean of positive samples being 15.4 x 10^4^ cells/g. (Limit of detection for automated counting is 10^6^ cells/g). These cell counts were too low to allow formal diversity analysis, however a representation of bacterial composition in positive samples is shown in Fig 1. The colonisation of a single baby was dominated by *Enterobacteriaceae*. The others contained 2–5 genera. *Bifidobacterium*, *Enterobacteriaceae*, *Enterococcaceae* and *Bacteroides*-*Prevotella* were the most abundant bacteria identified.

**Fig 2:**
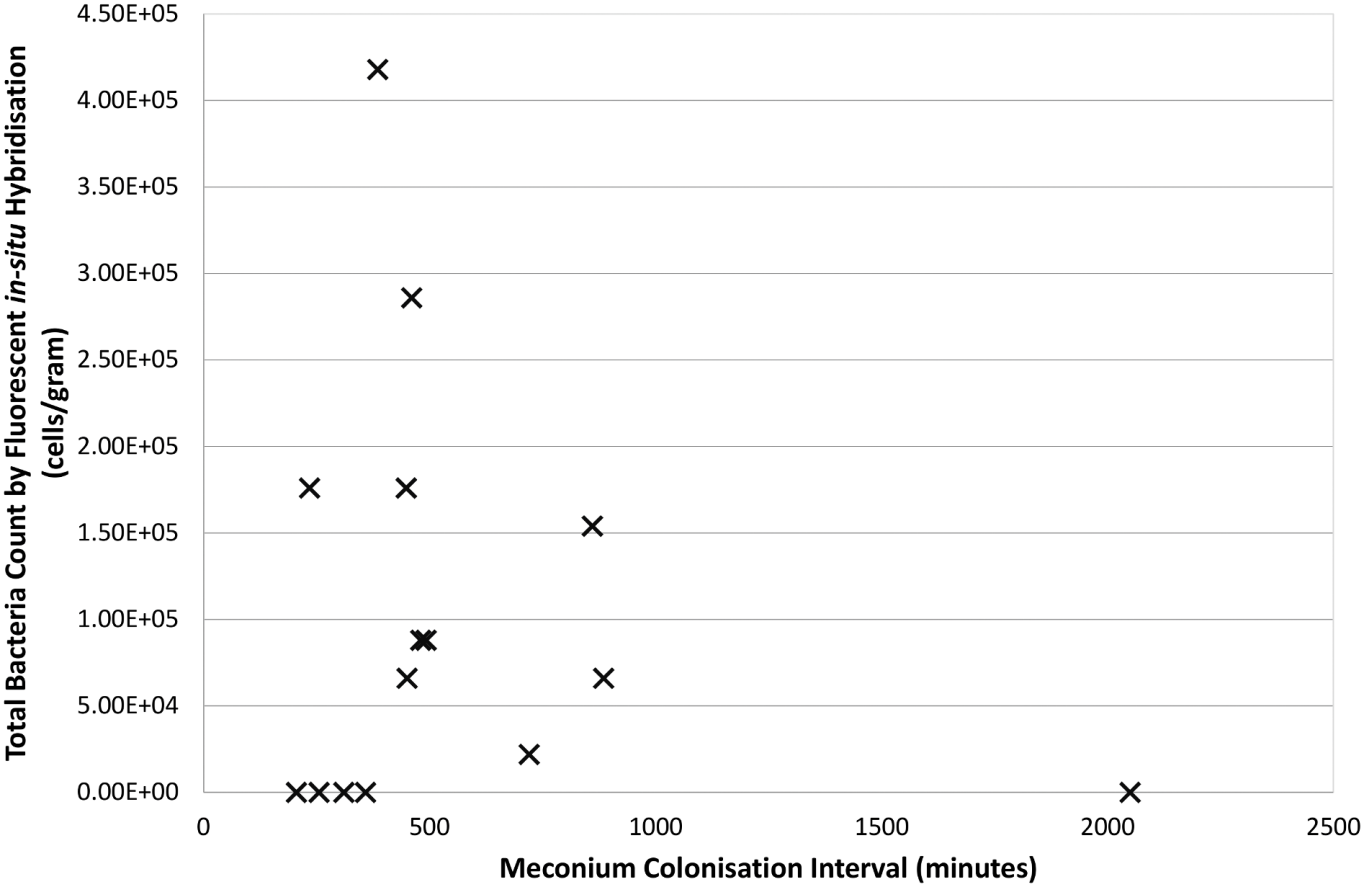
XY Scatterplot of “meconium colonisation interval” (defined as rupture of membrane interval plus time to passage of meconium plus sample time to laboratory) against total bacterial count (r^2^ = 0.023).

PCR amplification using generic bacterial primers was only possible in DNA extracted from one sample. The remaining 9/10 FISH positive samples failed to give any amplification products, despite positive spiked samples demonstrating the feasibility of a reaction. There was no clear association between “meconium colonisation interval” (MCI) with bacterial counts (Fig 2, Pearson correlation r^2^ = 0.023), however interestingly four of seven samples with an MCI <500 minutes were sterile compared with only one of eight above this threshold.

## Discussion

We designed the current study, with tight clinical criteria looking to identify healthy infants, born vaginally to healthy mothers and with clinical parameters as close to normal physiology as possible. We deliberately put in place tight inclusion/exclusion criteria and were firm in rejecting samples that did not meet these. Despite this, we have demonstrated evidence of bacteria, albeit with very limited cell counts and diversity, in first-pass meconium samples. In attempting to look for a temporal relationship to colonisation, we defined the “meconium colonisation interval” (MCI) as the period between externalisation of the amniotic sac contents (by rupture of membranes) and processing of the meconium sample in the laboratory. Assuming sterility of the amniotic sac and fetus, the start of this period would coincide with the commencement of fetal microbial colonisation. We demonstrated no correlation between MCI and bacterial cell counts in this study, albeit with limited subject numbers and consequently limited power to demonstrate such an association. Around one third of first-pass meconium samples in our cohort appeared sterile on triplicate microscopy analysis by FISH, and four of these five samples had an MCI time <500 minutes. No meconium sample was obtained in this study with an MCI time <200 minutes (3 hours 20 minutes), a considerable gap during which microbial changes may well occur. Addressing the MCI in the question of gut sterility at birth is a challenge for studies in this field and we would suggest that recording these times and analysing microbial data using MCI may help in future studies exploring early colonisation. This is a particular difficulty when addressing premature neonatal colonisation as delayed passage of meconium is commonplace with prematurity and often takes a number of days [14]. Microbial diversity has been reported in meconium samples from premature infants, though with scant consideration given to the timing of such samples with regards other early-life colonisation events [15–18].

Comparing our study against other such investigations in the term neonate is challenging, as most utilise a “catch-all” approach to recruitment, rather than our selective approach, and presume all first pass samples are equivalent. The genre-defining culture-based paper on this subject from Jiménez and colleagues looked at early-passed meconium (within 2 hours of birth) and processed half of these immediately and half within 4 days [1]. No data was given on rupture of membranes interval. There was a clear distinction between the two groups with more culture-recovery in those with delayed processing. Between 1 and 5 species were isolated per sample with a predominance of *Enterococcus faecalis*, *Staphylococcus epidermidis* and *Escherichia coli*. No sample was entirely sterile. Makino and colleagues recently demonstrated a dearth of *Bifidobacteria* colonisation in first-pass meconium, but did not extend the analysis beyond this single genus [19]. Hu and colleagues performed a comprehensive 16S rDNA amplicon sequencing study on first-pass meconium samples from 23 newborns, including 10 from mothers with gestational or type II diabetes and 13 with no maternal diabetes [20]. The extremes of range for times of meconium passage was given (2–48 hours), but no data on rupture of membranes interval or time to lab processing. All samples had DNA evidence of bacteria, with a large Proteobacteria abundance in the infants without diabetic mothers. Ardissone *et al* recently published a further amplicon sequencing study on meconium samples from both term and preterm infants, and could find no DNA evidence of bacteria in 8/17 (47%) of meconium from infants >33 weeks gestation, with a greater proportion of preterm infants positive (74% vs 53%) [15]. Positive samples were extremely limited in diversity, much as in our study, with dominance by a single genus common. Specific data on MCI derivative periods was not provided, but it would be interesting to see whether delayed passage of meconium contributed to the increased positivity seen in premature infants.

In summary, we present here a clinically considered study on a select group of infants, selected for “normality” as much as possible, demonstrating that although ~2/3 of first-pass meconium samples contain bacteria, this appears less common when colonisation factors and timings are considered, and the number of different bacterial species detected in positive samples appears extremely limited. Further targeted work on the earliest aspects of microbial colonisation is required to fully address the sterility question, and we propose use of the MCI and its constituent timings as a potentially important aspect of data gathering for such studies. The seminal study to date on early neonatal colonisation demonstrated a lack of differentiation across neonatal body sites and inferred that this was a feature of early colonisation [21]. The impact of pioneer organisms may well influence downstream colonisation [22], and future attempts to influence the neonatal microbiota for the benefit of long-term health hinge on our understanding of early colonisation.
